# Population type influences the rate of ageing

**DOI:** 10.1038/s41437-019-0187-1

**Published:** 2019-02-08

**Authors:** Andrew DJ. Overall, Richard GA. Faragher

**Affiliations:** School of Pharmacy and Biomolecular Sciences, Huxley Building, University of Brighton, Brighton, East Sussex BN2 4GJ UK

**Keywords:** Evolutionary genetics, Population genetics

## Abstract

Mutation accumulation is one of the major genetic theories of ageing and predicts that the frequencies of deleterious alleles that are neutral to selection until post-reproductive years are influenced by random genetic drift. The effective population size (*N*_*e*_) determines the rate of drift and in age-structured populations is a function of generation time, the number of newborn individuals and reproductive value. We hypothesise that over the last 50,000 years, the human population survivorship curve has experienced a shift from one of constant mortality and no senescence (known as a Type-II population) to one of delayed, but strong senescence (known as a Type-I population). We simulate drift in age-structured populations to explore the sensitivity of different population ‘types’ to generation time and contrast our results with predictions based purely on estimates of *N*_*e*_. We conclude that estimates of *N*_*e*_ do not always accurately predict the rates of drift between populations with different survivorship curves and that survivorship curves are useful predictors of the sensitivity of a population to generation time. We find that a shift from an ancestral Type-II to a modern Type-I population coincides with an increase in the rate of drift unless accompanied by an increase in generation time. Both population type and generation time are therefore relevant to the contribution mutation accumulation makes to the genetic underpinnings of senescence.

## Introduction

**Fig. 1:**
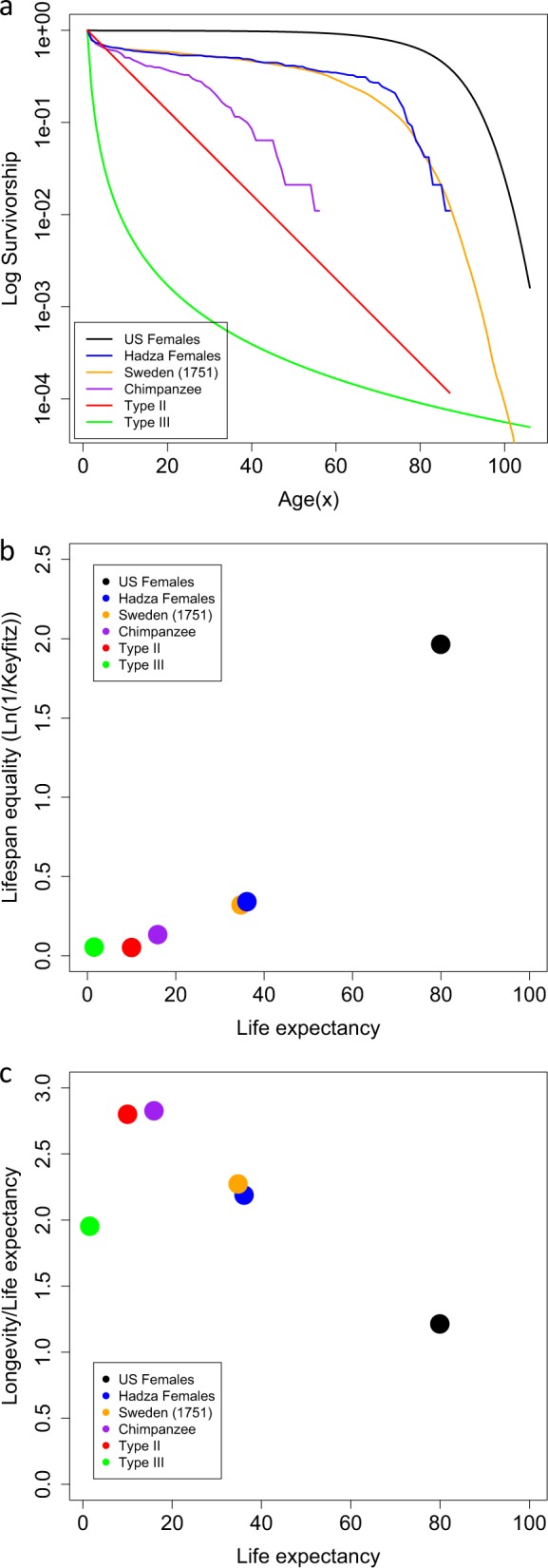
**a** Comparison of survivorship curves for six populations. The US females from 2000 (black, Templeton 2006) represent a Type-I population. The Swedish females from 1751 (orange, Human Mortality Database (http://www.mortality.org)) and Hadza females (blue, Blurton Jones 2016) represent Type-I populations prior to the increased longevity seen in modern populations. The wild Chimpanzee (purple, Bronikowski et al. 2016) represents our closest living species. The Type-II population (red, simulated (see Methods for details)) is used as a close approximation of the chimpanzee population and the Type-III population (green, simulated (see Methods for details)) is used for completion as a comparator at the other end of the ‘type’ spectrum. **b** A continuum of lifespan equality (ln(1/*H*)) and life expectancy in six populations presented in **a**. **c** A comparison of ‘pace’ (life expectancy) and ‘shape’ (ratio of longevity to life expectancy) for six populations in **a**

The survivorship curve is a useful visualisation of the frequency distribution of the age classes of a population (Rauschert 2010) and is calculated as *l*_*x*_ = *n*_*x*_/*n*_0_, where *n*_*x*_ is the number of individuals in the study population who survive to the beginning of age category *x* and *n*_0_ is the number of newborns. If the population is stable, then survivorship curves describe how the numbers of individuals of a cohort decline with time. When the logarithm of the number of survivors is plot against age, then three distinct, idealized “types” are distinguished (Type-I, Type-II and Type-III (Deevey 1947); Fig. 1a). Survival curves, by deduction, give some indication of the rate at which mortality increases with age and, therefore, the rate of senescence of the population. It has also been stated that variation in survival curves reflects species sensitivity to the genetic and environmental factors that have shaped their evolutionary history (Demetrius 2013). The survival curve of modern humans is described as a classic “Type-I”, where the probability of survival is high until relative old age, whereby it then declines rapidly, which is typical of many large mammals. A Type-I survival curve is also typical of pre-industrial human populations (e.g., the 1751 Swedish population in Fig. 1a) and ancient societies, such as hunter-gatherer, forager-horticulturalist and acculturated hunter-gatherer societies, a modern example being the indigenous Hadza population of Tanzania (Gurven and Kaplin 2007, Fig. 1a). Our closest living relative species, the chimpanzee, has a survivorship curve that is variable, depending on whether the population is wild or captive (Thompson et al. 2007), but the wild examples are arguably closer to a Type-II (Hill et al. 2001; Bronikowski et al. 2016), which is described by a constant proportion of individuals dying over time. For illustrative purposes we generated an example Type-II population with a 10% probability of mortality from one age class to the next, which aligns closely with the chimpanzee population (Fig. 1a). Ancestral human populations studied from archaeological specimens, for example the Libben site skeletal sample, which is radiocarbon dated to between 800 and 1100 CE, have been described as Type-II populations (Lovejoy et al. 1977). However, the remains from such sites are not always representative of the total population (Howell 1982); hence, survivorship curves derived from archaeological specimens are not considered in this study. By comparison, Type-III populations display very high mortality at young ages, but those that do survive to adulthood go on to have a relatively long life expectancy, which is typical of tree and insect species. The difference between the Type-I survivorship curves of hunter-gatherer and pre-industrial populations and the Type-I survivorship curve of modern industrial populations is difficult to quantify and identifies a limitation of this approach when considering the evolutionary demography of a species.

An early approach to quantifying the information contained within survivorship curves was to use its logarithm, for example Keyfitz’s entropy, $H = \frac{{ - \mathop {\smallint }\nolimits_0^\infty \left( {{\rm ln}l_x} \right)l_xdx}}{{\mathop {\smallint }\nolimits_0^\infty l_xdx}} = - \frac{{e^\dagger }}{{e_0}}$ (Keyfitz 1977, Goldman and Lord 1986), described as the ratio of life expectancy lost due to death (*e*^†^) to that of life expectancy (*e*_0_). This more quantitative approach to describing age-distributions has recently been developed to distinguish between the ‘pace’ and ‘shape’ of change of populations as they age (Baudisch 2011; Baudisch et al. 2013). The ‘pace’ of life, or how fast populations age, can be measured as life expectancy (*e*_0_), which captures the average length of life, or the tempo at which organisms survive and reproduce, placing organisms on a fast/slow continuum of ageing (Baudisch 2011; Colchero et al. 2016). On the other hand, the ‘shape’ describes the direction and degree of change in mortality (Wrycza et al. 2015) and hence captures the rate at which a species senesces (Baudisch 2011). There are various ways of measuring the ‘shape’ of ageing, all of which are highly correlated (Wrycza et al. 2015). Figure 1b, c illustrates two methods of measuring ‘shape’; one is generally referred to as life table entropy (sometimes lifespan equality), which can be calculated as ln(1/Keyfitz’s entropy) (Wrycza et al. 2015, Colchero et al. 2016). Another is the ratio of longevity (Ω)/life expectancy (*e*_0_), where Ω is the age at which, for example, 95% of the adults have died. Although there are many measures (Wrycza et al. 2015), we present these two as they have previously been used for cross-species comparisons (Baudisch 2011; Colchero et al. 2016). As can be seen from Fig. 1, there is some correspondence between these pace/shape metrics and the survivorship curves: the wild chimpanzee population clusters with the Type-II example; the pre-industrial/hunter-gatherer Type-I populations cluster together and the modern US Type-I population stands apart from all of these. For the populations considered here, life expectancy increases from Type-III to Type-II to Type-I, as expected, and shows that population Type corresponds more obviously with notions of the ‘pace’ of life. There are, however, some important subtleties. For example, the distinct Type-II and Type-III survivorship curves presented in Fig. 1a have similar ‘shape’ (Fig. 1b, c). Because Type-II populations show constant mortality with age, they are representative of populations that show negligible senescence. Baudisch (2011) points out that long-lived species typically present negligible senescence, for example, long-lived trees and marine invertebrates mostly show Type-III survivorship. For this reason Type-II and Type-III populations should cluster on the ‘shape’ axis. However, there are clear exceptions to this rule, modern human populations being one as, although they are long-lived, they have a shape score indicative of strong senescence. As helpful as pace and shape are at capturing important aspects of a species life-history, we believe they do not lend themselves so readily to visual comparisons of survival/mortality with age where the corresponding reproductive distribution is being studied. Here we explore the influence of the reproductive distribution, specifically generation time, on the rate of genetic drift for human populations, with current and hypothetical ancestral survival curves. The aim is to identify the influence of population ‘type’ and generation time on mutation accumulation and, as a consequence, the corresponding changing role of mutation accumulation on the senescence of these populations.

The current longevity that modern humans experience came late in human evolution, where for the first time during the Upper Palaeolithic (~50,000 to 10,000 years ago) there are a larger number of older adults amongst the deceased than there are younger adults (Caspari and Lee 2004). Evidence from throughout the late Archaic up to the Upper Palaeolithic also indicates that mortality patterns for young (pre- 40 years) versus old (post- 40 years) did not alter during this period and that older individuals would have been rare (Trinkaus 2011). If the wild chimpanzee/Type-II survivorships are indicative of our common *ancestral* type, then judging by modern hunter-gatherer societies and the evidence from Upper Palaeolithic humans (Caspari and Lee 2004), it seems reasonable to assume that the Type-I distribution is generally reflective of human populations since the Neolithic period (~500 generations ago). This has implications for the evolution of ageing in human populations as well as the laboratory models used to study it. We hypothesise that a Type-II survivorship curve displaying a constant mortality rate and relatively few older reproductive adults describes the majority of our ancestral demography, up until the Palaeolithic. A Palaeolithic, demographic shift towards the modern Type-I survivorship curve would then have followed this, with the older, parental age classes now numerically better represented. The greatest shift in modern human evolution may not simply be one of increasing population size, but rather a change in the survivorship curve (Type-II to Type-I), which has implications for the genetic drift of deleterious mutations, and hence mutation accumulation.

Standard population genetics theory tells us that the allele frequencies of mutations entering small populations will drift to a greater degree (greater stochasticity) than those entering large populations, as genetic diversity is lost at a rate proportional to 1/2*N*_*e*_, where *N*_*e*_ is the effective population size. A mutation with a negative impact on the fitness of the homozygous genotype (e.g., aa) relative to the wild-type homozygote (e.g. AA), measured as *s*, is effectively removed by natural selection whenever *s* > 1/ 2*N*_*e*_, else drift dominates the rate of loss (Hartl and Clark 2007). If we consider a snapshot of a population with overlapping generations, then we expect to see a monotonic decline in the number of individuals with increasing age due to the unavoidable causes of mortality that individuals encounter with the passing of time. This then leads to a proportional decline in the effectiveness of selection with age due to the relatively few individuals of older age contributing to the genetics of future generations, known as Hamilton’s principle (Hamilton 1966). Because selection removes mutations more effectively when they have a detrimental phenotypic effect early on in life compared with mutations that only affect older individuals, detrimental mutations accumulate (Medawar 1952; Charlesworth and Williamson 1975). Importantly, the negative affect of these mutations may persist in post-reproductive ages, contributing to the ageing phenotype.

The effect of drift on late-acting deleterious mutations is one of potential inflation of their frequencies, where older age-classes suffer a greater loss of health relative to the younger age classes and hence where smaller populations age at a faster rate than larger populations. Lohr et al. (2014) explicitly tested this hypothesis using *Daphnia magna* and, consistent with other recent work (Jones et al. 2008) identified a correlation with age at first reproductive output and rate of ageing across numerous wild and model organisms, which is consistent with the expectation that populations with low genetic diversity have accelerated ageing. The main conclusion of Jones et al. (2008) is that the onset and rate of senescence in both survival and reproduction are associated with generation time, an aspect that is not usually considered with the ‘pace’ or ‘shape’ of ageing in populations. Given that generation time influences *N*_*e*_ (Waples and Yokota 2007), where generally *N*_*e*_ ∝ *N*_*nb*_*T*, where *N*_*nb*_ is the number of newborns arriving in each generation and *T* is the generation time (mean age of parents), then we expect effective population size to decline with shorter generation times. It then becomes conceivable that the age of sexual maturity, as well as the survivorship and reproductive span of a species, have consequences for the role of mutation accumulation in ageing phenotypes.

The phenotypic effect of a mutation entering a population is dependent upon when the gene is expressed, which can be age-specific (e.g., developmental genes, genes associated with sexual maturity and female menopause), although not always, as when a gene’s influence can be cumulative (e.g., IL1RAP and its influence on amyloid plaque accumulation (Ramanan et al. 2015)). When the mutation has an age-specific expression, its survival depends upon the size of this age class. As a population declines in size, the age classes that are large enough for selection to dominate drift (i.e., *s* > 1/2*N*_*e*_) will shift towards the younger classes. Mutations that have a detrimental effect on survival and reproduction are therefore more likely to persist in the older age classes.

According to traditional ecology theory, population density governs the optimal age of maturity (Macarthur and Wilson 1967), distinguishing *r*-selected species (typically small organisms producing many offspring, with early maturity and with short lifespans) from *K*-species (reproduce slowly at later ages and with longer life-spans) (Pianka 1970). Most primate species, including humans, are *K*-species. This *r*/*K* categorisation can be quite sensitive to environmental factors. For example, an increase in environmental stochasticity can select for an *r*-type strategy (Engen and Saether 2016). Although this *r*/*K* categorisation of life histories persists in some of the ageing literature (e.g., Reichard 2016), there is now a preference for placing species on a fast–slow continuum, or tempo, of life history. One method is to use the ratio of fertility rate to age at first reproduction, which among other factors is less sensitive to environmental stochasticity (Oli 2004). Which of these *r*/*K* strategies, or position on the fast–slow continuum, a species adopts may be highly constrained by their environment and evolutionary history. Nevertheless, we hypothesise that there will have been situations, e.g., during the Neolithic period and modern industrialization, where the change in culture, environment and reproductive patterns on human survival would have been of sufficient magnitude that the species survivorship curve shifted, resulting in consequent changes in both the ‘pace’ and ‘shape’ of their life history. The consequence is that, once a modern-industrialised life-history strategy emerged, the older reproductive age classes once poorly represented in a Type-II population and potentially subject to the consequences of mutation accumulation, become numerically better represented in a Type-I population. Using simulations, we explore the significance of this shift on the rate of genetic drift and the contribution mutation accumulation is likely to make to the ageing, or senescence, of the population. The value in doing so is that any insights relating to the magnitude of influence past demographic shifts have had on the present evolution of ageing in the human population would better inform the choice of model organism employed in its study.

## Methods

Felsenstein’s method of estimating the effective population size of an age-structured population (Felsenstein 1971, equation 10) was used to measure the influence of generation time on the rate of drift across populations with Type-I and Type-II survival curves:

1 $$N_e = \frac{{N_{nb}T}}{{1 + \mathop {\sum }\nolimits_{x = 1}^k l_xs_xd_xv_{x + 1}^2}},$$

where, for *k* age classes, *N*_*nb*_ is the number of newborns, *d*_*x*_ = *l*_*x*_ – *l*_*x*+1_ and *v* the reproductive value: $v_x = \mathop {\sum }\limits_{i = 1}^k l_im_i/l_x.$ Because our focus was the hypothetical shift in population type during human evolution, Type-III populations were not considered in any further detail. Following convention, we consider the female constituents of a population of parents and offspring. The number of females of each age (*x*) is denoted as *n*_*x*_ (equivalent to the number of females that survive each age class, *s*_*x*_), with the fecundity of each age class denoted as *m*_*x*_. The probability of surviving *to* each age class from birth is *l*_*x*_, which is simply *l*_*x*_ = *n*_*x*_*/n*_0_. The probability of surviving each age *x* is *P*_*x*_ = *l*_*x*_*/l*_*x*−1_. By convention, *l*_0_ = 1. For simplicity, we consider sampling the number of recruits for the next generation post-breeding. With a birth-pulse model, the effective fecundity of females of age *x* is *f*_*x*_ = *P*_*x*−1_.*m*_*x*_. The net reproductive rate of the population, per generation, is $R_0 = \mathop {\sum }\nolimits^ l_xm_x$. Importantly, *R*_0_ is the change in population size by *generation*, *T*, where $T = \frac{{\mathop {\sum }\nolimits_{x = 0}^k xl_xm_x}}{{\mathop {\sum }\nolimits_{x = 0}^k l_xm_x}}$, and where *k* is the total number of age classes. Hence, the intrinsic rate of population growth is $r = \frac{{{\rm ln}R_0}}{T}$. Importantly for this discussion, this shows clearly that as *T* increases, the intrinsic rate of population growth (*r*) decreases, hence, for population sizes to remain stable, as they do for our simulations, a change in *T* requires a compensatory change in *m*_*x*_.

In order to discriminate between the effect of young and old breeders on the loss of genetic diversity, we explicitly explore the influence of generation time and survivorship by employing a Leslie matrix approach. We use three example survivorship curves to illustrate the influence they may have on the rate of genetic drift. Life history data were obtained from 1) the US female population census from 2000 (Templeton 2006), considered to be typical of a Type-I population where there is high survival until late in life. 2) The Hadza female population (modelled by Blurton-Jones 2016) and, for comparison, 3) a fictional population representative of a typical Type-II population with a constant 10% mortality rate over yearly age classes: *l*_*x*+1_ = 0.9 × *l*_*x*_, designed to be similar to a wild chimpanzee population (Fig. 1a). The age classes (*x*) are 1 year apart. A Type-III population was also generated for comparison in Fig. 1 as *l*_*x*_ = *x*^–^^2.126^. The fecundity schedule, which is the tabulation of birth rates (*m*_*x*_), is manipulated similarly to Ryman (Ryman 1997), where the fecundity of each age class of each population is adjusted such that $\mathop {\sum }\nolimits^ l_xm_x = 1$. Here our focus is on the variability in survival curves, so the fecundity trajectories for Hadza and Type-II populations are a manipulation of the US female trajectory. The US female fecundity schedule is taken from Templeton (2006) and simulated as a normal distribution with mean = 27 years and variance = 7 years. The simulated populations have stable age-structure and constant size.

Assuming the age-specific survival and fecundity probabilities remain constant across generations (*t*), we track the number of females (*n*_*x*_), starting with age class *x*+1, by multiplication with a Leslie matrix for 100 generations to reach a stable age distribution. We then track the frequency of two alleles, *A* and *B*, at a single locus over a 1000 year time period by multiplying our Leslie matrix **L**, by a mating matrix **M**, such that **n**(*t* *+* *1*) = **LMn**(*t*) (Roughgarden 1998):

$$\begin{array}{*{20}{l}} {\left( {\begin{array}{*{20}{l}} {n_{_t + 1,x,AA}} \hfill \\ {n_{_t + 1,x,AB}} \hfill \\ {n_{_t + 1,x,BB}} \hfill \\ {n_{_t + 1,x + 1,AA}} \hfill \\ {n_{_t + 1,x + 1,AB}} \hfill \\ \vdots \hfill \\ {n_{_t + 1,k - 1,BB}} \hfill \end{array}} \right)} \hfill & = \hfill & {\left( {\begin{array}{*{20}{l}} {f_{x,AA}} \hfill & {f_{x,AB}} \hfill & {f_{x,BB}} \hfill & {f_{x + 1,AA}} \hfill & {f_{x + 1,AB}} \hfill & \cdots \hfill & {f_{k - 1,BB}} \hfill \\ 0 \hfill & 0 \hfill & 0 \hfill & 0 \hfill & 0 \hfill & \ldots \hfill & 0 \hfill \\ 0 \hfill & 0 \hfill & 0 \hfill & 0 \hfill & 0 \hfill & \ldots \hfill & 0 \hfill \\ {P_{x,AA}} \hfill & 0 \hfill & 0 \hfill & 0 \hfill & 0 \hfill & \ldots \hfill & 0 \hfill \\ 0 \hfill & {P_{x,AB}} \hfill & 0 \hfill & 0 \hfill & 0 \hfill & \ldots \hfill & 0 \hfill \\ \vdots \hfill & \vdots \hfill & \vdots \hfill & \vdots \hfill & \vdots \hfill & \ddots \hfill & \vdots \hfill \\ 0 \hfill & 0 \hfill & 0 \hfill & 0 \hfill & 0 \hfill & {P_{_k - 1,BB}} \hfill & 0 \hfill \end{array}} \right)} \hfill \\ {} \hfill & {} \hfill & { \times \left( {\begin{array}{*{20}{l}} {p^2} \hfill & 0 \hfill & 0 \hfill & 0 \hfill & 0 \hfill & \ldots \hfill & 0 \hfill \\ {2_{pq}} \hfill & 0 \hfill & 0 \hfill & 0 \hfill & 0 \hfill & \ldots \hfill & 0 \hfill \\ {q^2} \hfill & 0 \hfill & 0 \hfill & 0 \hfill & 0 \hfill & \ldots \hfill & 0 \hfill \\ 0 \hfill & 0 \hfill & 0 \hfill & 1 \hfill & 0 \hfill & \ldots \hfill & 0 \hfill \\ 0 \hfill & 0 \hfill & 0 \hfill & 0 \hfill & 1 \hfill & \ldots \hfill & 0 \hfill \\ \vdots \hfill & \vdots \hfill & \vdots \hfill & \vdots \hfill & \vdots \hfill & \ddots \hfill & \vdots \hfill \\ 0 \hfill & 0 \hfill & 0 \hfill & 0 \hfill & 0 \hfill & \ldots \hfill & 1 \hfill \end{array}} \right) \times \left( {\begin{array}{*{20}{l}} {n_{t,x,AA}} \hfill \\ {n_{t,x,AB}} \hfill \\ {n_{t,x,BB}} \hfill \\ {n_{t,x + 1,AA}} \hfill \\ {n_{t,x + 1,AB}} \hfill \\ \vdots \hfill \\ {n_{t,k - 1,BB}} \hfill \end{array}} \right)} \hfill \end{array}.$$

As described in detail in (Roughgarden 1998), the number of newborn females at time *t*+1 is $b_{t + 1} = \mathop {\sum }\nolimits_{x,ij} f_{x,ij}n_{t,ij}$ for alleles *i, j*. The allele frequencies amongst the newborn are simply the result of Mendelian segregation:

$$p_{t + 1} = \frac{{\mathop {\sum }\nolimits_x f_{x,{\rm AA}}n_{x,{\rm AA}} + \left( {1/2} \right)\mathop {\sum }\nolimits_x f_{x,{\rm AB}}n_{x,{\rm AB}}}}{{b_{t + 1}}}.$$

This describes the random union of gametes between parents across all age classes and maintains Hardy-Weinberg genotypic ratios among the newborn, presented in the mating matrix **M**. We performed all calculations and iterations of the matrices in *R* (R Development Core Team 2008). The R Script and input data are available from https://github.com/AndyOverall/DriftAgeStruct, along with GNU public license details.

The simulation of genetic drift is a two-stage process. The first deals with the random union of gametes to generate the newborn class. The second models the random culling of alleles with time such that the allele frequency distribution in the breeder’s age class (e.g., *n*_*t=20, x=20*_) is a random subsample of the allele frequency distribution of the age class when they were newborns (*n*_*t=1, x=1*_). The number of newborn individuals is kept constant and takes the value of the first age class of the stable age distribution (i.e., the one resulting from 100 iterations of the Leslie matrix multiplication: **n**(*t* = 100) = **Ln**(*t* = 99)). For example, considering the US Female population, where $\mathop {\sum }\nolimits_{x = 1}^k n_{100,x} = 1000$, the number of newborns (age class *x* = 1) = *n*_100,1_ = 13, with the numbers of genotypes being in accord with Hardy-Weinberg proportions: *n*_100,1,AA_ + *n*_*1*00,1,AB_ + *n*_100,1,BB_ = 13. The simulation of random genetic drift involves the random sampling of these 13 genotypes using the *R* function **rmultinom**, **rmultinom**(n, size, prob). If *X* is a random variable such that *X* = {*n*_AA_, *n*_AB_, *n*_BB_}, then **X** = **rmultinom**(1,13,*n*_AA_/13,*n*_AB_/13,*n*_BB_/13) regenerates the newborn’s genotypes subsequent to drift and from these genotypes the new allele frequencies are obtained. These frequencies then feed directly into the mating matrix **M**. If *P*_*x*_ and *m*_*x*_ remain constant, iterations of this procedure simulate the frequency of the alleles under the influence of drift. The simulation included 1000 repeats, to model 1000 reproductive events, each iterated 1000 times to correspond with the passage of 1000 years.

In addition to allele frequency stochasticity occurring in the generation of newborn’s genotypes, there is a random cull of genotypes between age classes over generations. For example, with neutral alleles, the number of genotypes of age class *x* = 2 in generation 1 (*n*_*1,2*,AA_; *n*_*1,2*,AB_ and *n*_*1,2*,BB_) each has a probability (*P*_x_) of surviving to the next generation (*n*_*2,2*,AA_; *n*_*2,2*,AB_ and *n*_*2,2*,BB_). This is simulated, in this example, by *n*_*2,2,ij*_ = *P*_1_ × *n*_*1,2,ij*_. Then, *P*_x_% of *n*_*2,2,ij*_ remain the same genotypes, with the remaining 1–*P*_x_% being randomly drawn using the same *R* function **rmultinom**.

The simulation of selection involved a modification of the survival probability. For example, for neutral alleles, the survival of the genotypes from one age class (*x*) to the next is equal (e.g., *P*_*x*,AA_ = *P*_*x*,AB_ = *P*_*x*,BB_). Negative selection for individuals that are homozygous for recessive alleles is simulated as *P*_*x*,BB_ < *P*_*x*,AA_, *P*_*x*,AB_.

**Fig. 2:**
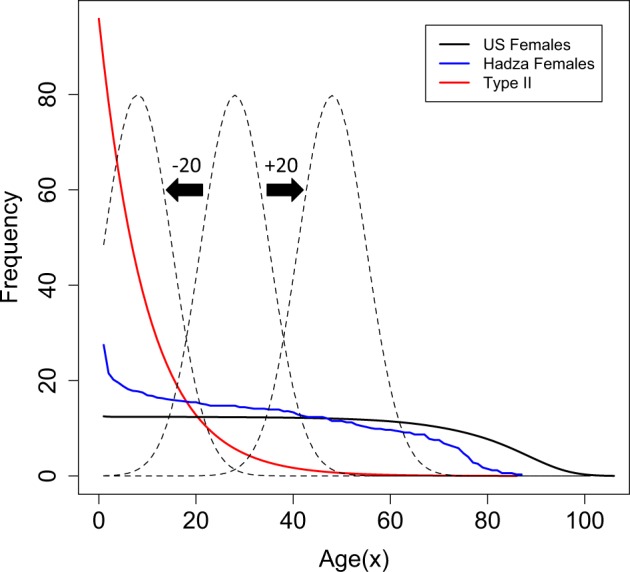
Survivorship curves for three populations: US Female population from 2000, Hadza females and a Type-II population with survivorship of *l*_*x*+1_ = 0.9 × *l*_*x*_. Age (*x*) is in years. Central dashed curve shows the reproductive distribution of the US female population, where the frequency scale on the *y*-axis is arbitrary. Left hand curve shows the reproductive distribution shifted 20 years earlier (*T*−20) and the right hand curve shows the reproductive distribution shifted 20 years later (T+20)

Figure 2 illustrates the survival curves for the three populations where the total population size is *N* = 1000. The middle dashed curve shows the reproductive distribution for the US female population (where the *y*-axis frequency scale is arbitrary), which corresponds to a generation time $T = \frac{{\mathop {\sum }\nolimits_{x = 0}^k xl_xm_x}}{{\mathop {\sum }\nolimits_{x = 0}^k l_xm_x}} = 27$. Shifting the reproductive distribution either towards younger or older age classes modified the generation times. For example, the dashed curve of Fig. 2 furthest to the left shows the reproductive distribution shifted 20 years younger and, for the US females, *T* = 8.8 (referred to as T-20). When shifted 20 years towards the more elderly individuals, shown by the dashed curve to the right, *T* = 46.8 (referred to as *T* + 20). This manipulation separates out the influence of young versus older breeders, whilst acknowledging that the corresponding plausibility of such an early/late age of reproduction may be unrealistic.

## Results

**Fig. 3:**
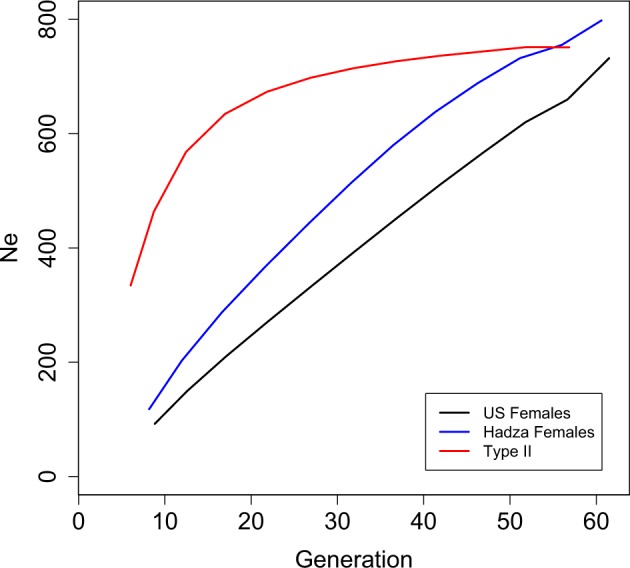
The change in *N*_*e*_ with generation time for three population types: US Female population from 2000, Hadza females and a Type-II population with survivorship of *l*_*x*+1_ = 0.9 × *l*_*x*_. Each population has a total population size of *N* = 1000. Generation = generation time calculated as $= \frac{{\mathop {\sum }\nolimits_{x = 0}^k xl_xm_x}}{{\mathop {\sum }\nolimits_{x = 0}^k l_xm_x}}$

Setting *N* = 1000 for each of the three populations, Felsenstein’s estimate of *N*_*e*_ (Eq.1) results in a simple linear increase in *N*_*e*_ with generation time (*T*) for both Type-I populations (US and Hadza females), but plateaus for the Type-II (Fig. 3). This comes as no surprise for both Type-I populations as the survivorship curve barely changes over the age range considered here (10–60 years), and so *N*_*e*_ ∝ *T*, as the other parameters in Eq. 1 change very little with increasing *T*. However, for the Type-II population, after an initial increase in *N*_*e*_ with *T*, the relationship plateaus and *N*_*e*_ becomes largely independent of generation time as a corresponding increase in reproductive value (*v*_*x*_) balances the increase in *N*_*e*_ with *T* (results not shown).

**Fig. 4:**
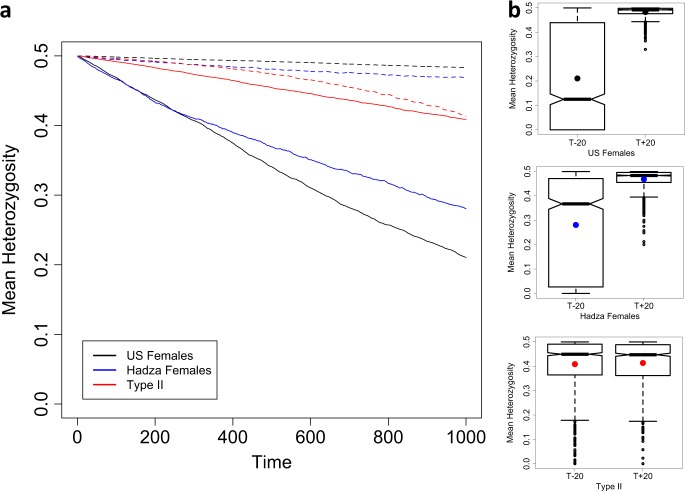
**a** Mean decline in heterozygosity from 1000 age-structured simulations where the starting frequency is *P*_*B*_ = 0.5, *N* = 1000, where reproductive distributions have been shifted towards the young (*T*−20, solid lines) and the old (*T*+20, dashed lines). Time is in years. **b** Notched boxplots summarising each of the 1000 simulations run for each population. The black, blue and red dots indicate the means for the US, Hadza and Type-II populations, respectively

The expected loss-of-heterozygosity (*H*) over time is a function of *N*_*e*_, e.g., $H_{t + 1} = H_t\left( {1 - \frac{1}{{2N_e}}} \right)$ (Gillespie 2004). However, for age-structured populations, the number of newborns (*N*_*nb*_) and reproductive values (*v*_*x*_), as well as generation time influence estimates of *N*_*e*_. Although Felsenstein (and others, e.g., (Hill 1972) have formulated estimates of *N*_*e*_ for age-structured populations, it is not immediately obvious how this translates to a loss of heterozygosity over time. For *N* = 1000, the estimate of *N*_*e*_ is larger for the Type-II population than Type-I populations across the generation times considered here (Fig. 3), leading to the expectation that drift would proceed at a slower rate for the Type-II population. Figure 4a shows the decline in mean heterozygosity over time for populations of early (*T* − 20) and late (*T* + 20) breeding individuals across the three populations. Figure 4b presents notched boxplots to summarise the distribution of heterozygosity values at the final time point (Time = 1000) for 1000 replicates. The “notch” of the boxplots span the 95% confidence interval of the median and the box itself spans the interquartile range. Informally, if the notches do not overlap, then it is considered that the medians differ with 95% confidence. These plots show that for the Type-I populations, the earlier breeding populations lose heterozygosity, on average, at a markedly greater rate than later breeding populations and that these earlier breeding populations are highly variable in the rate of this loss. However, for Type-II populations, the distribution of heterozygosity values for populations with early breeders is indistinguishable from that of late breeding populations. For early breeding populations (T−20), the rate of loss is consistent with the Felsenstein *N*_*e*_ estimates presented in Fig. 3: the smaller the *N*_*e*_, the greater the rate of loss of heterozygosity. However, Fig. 4a shows that for later breeding populations this is not the case and in fact the rate of drift is not accurately predicted from estimates of *N*_*e*_, with US females drifting the least and Type-II the most.

**Fig. 5:**
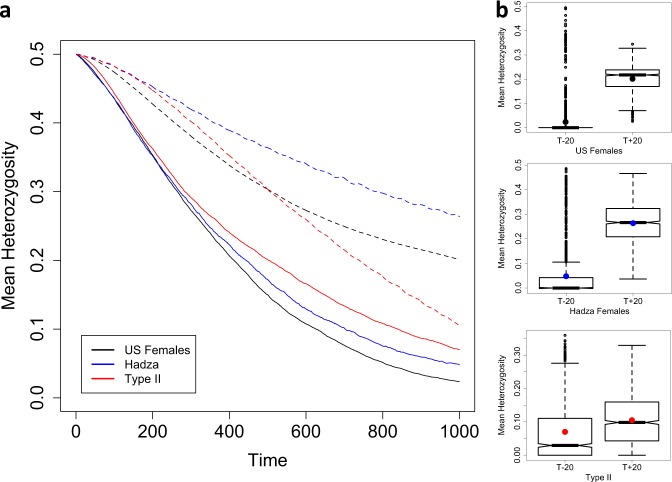
**a** Mean decline in heterozygosity from 1000 age-structured simulations where the starting frequency is *P*_*B*_ = 0.5, *N* = 1000, where reproductive distributions have been shifted towards the young (*T*−20, solid lines) and the old (*T*+20, dashed lines). Homozygous individuals (BB) have a reduced survival probability (*s* = 0.01). Time is in years. **b** Notched boxplots summarising each of the 1000 simulations run for each population. The black, blue and red dots indicate the means for the US, Hadza and Type-II populations, respectively

With the introduction of negative selection into the simulations, we expect to see heterozygosity lost at a greatest rate in populations, where *s* > 1/2*N*_*e*_ and to be independent of population type when s ≫ 1/2*N*_*e*_. When the recessive mutation reduces the survival of breeding individuals homozygous for this mutation (*P*_*x*,BB_) by 0.1% (*s*), the rate at which heterozygosity is lost is not altered noticeably (result not shown). However, Fig. 5a shows the decline in heterozygosity when *s* = 1% for early (*T* − 20) and late (*T* + 20) breeding populations where, as predicted, population type is still having some influence, more noticeably for the *T* + 20 populations where the distribution of heterozygosity values at time point *t* = 1000 are distinct between Type-I and Type-II populations (Fig. 5b).

## Discussion

The two main genetic causes of senescence, antagonistic pleiotropy and mutation accumulation, have different expectations with regard to the frequency distribution of mutations that are deleterious to the older age classes of a species (Rodrguez et al. 2016). Here we considered the consequence of population ‘type’ on the rate of drift in light of this influence on mutation accumulation. We hypothesised that the human survivorship curve has shifted relative to pre-industrialised and hunter-gatherer populations and possibly further back in time from ancestral populations that had survivorship curves more akin to chimpanzees. The literature on the theory underpinning estimates of *N*_*e*_, the rate-determining parameter of drift, is extensive (e.g., Engen et al. 2005), but there has been little attention paid towards the influence of population type (e.g., those that fit the ecological Type-I, II and III survivorship ideals). The potential importance of this lay in the fact that most ageing studies have an understandable bias towards human ageing, but the industrial-age human population type is unusual in being an extreme example of Type-I (see Fig. 1). Not only is this shift in population type likely to have consequences for the rate of mutation accumulation, there are also consequences relating to the appropriate choice of organism used to model the ageing of human populations. For example, a model organism’s evolutionary history shapes its life history, including its survivorship. Ancestral population type may then contribute to the genetic causes of the model organism’s senescence, which may be at variance with the evolutionary history of humans.

When Type-I and Type-II age-structured populations are of comparable size (e.g., *N* = 1000), then the number of newborns (*N*_*nb*_, age class *x* = 1) in Type-I populations are fewer than the *N*_*nb*_ of Type-II populations (Fig. 2). The relationship: *N*_*e*_ ∝ *N*_*nb*_*T* (Felsenstein 1971) predicts that Type-I populations will drift at a greater rate than Type-II populations. Figure 3 shows the results of applying Felsenstein’s estimate of *N*_*e*_ to the three population types showing that Type-I and Type-II populations differ in how sensitive these estimates of *N*_*e*_ are to changes in generation time, with the allele frequencies in Type-II populations drifting almost independently of generation time throughout the majority of the age range considered here. The reason for this is that the reproductive value of the breeding individuals (*v*_*x*_ in the denominator of Eq. 1) differs markedly between the young and elderly of a Type-II population relative to that of a Type-I. For the scenarios presented here, where populations are constrained to remain at a constant population size (e.g., *N* = 1000), an elderly breeder in a Type-II population is required to make a much greater contribution to the next generation in terms of offspring number, relative to an elderly breeder in a Type-I population. Hence, the increase in the reproductive value in Type-II populations balances the increase in *N*_*e*_ expected as a consequence of an increase in generation time (numerator of Eq.1), which is why the relationship between these two parameters (*T* and *N*_*e*_) weakens (Fig. 3).

Figure 4 shows the results of drift simulations for the populations presented in Fig. 3 (modern US females, Hadza females and a Type-II population). For the populations with an early generation time (T – 20, solid lines, Fig. 4a) the rate of drift corresponds with estimates of *N*_*e*_ presented in Fig. 3. However, for the populations with a later generation time (T + 20, dashed lines, Fig. 4), the mean rates of drift are the opposite of expectations based purely on the relative magnitudes of *N*_*e*_ (Type-II > Hadza females > US females), although the distributions of 1000 simulations performed here do overlap (Fig. 4b). It is not obvious what the cause of this reversal of mean heterozygosity value is. However, the ordering of the populations in terms of their rates of drift does appear to correspond with the number of breeders (*N*_Breeders_), indicated by the overlapping reproductive distributions in Fig. 2. For example, considering the T − 20 populations, the number of breeders throughout the reproductive distribution (dashed lines, Fig. 2) is ordered as Type-II > Hadza females > US females. However, the number of breeders throughout some of the T + 20 population’s reproductive distribution shows a reversed ordering. Taken together, the plots in Figs. 3 and 4a illustrate the magnitude of difference that population types have on the rate of drift and indicate a few subtleties that may be difficult to predict directly from Felsenstein’s estimate of *N*_*e*_. This outcome appears to be a simple consequence of the fact that, despite having equal census sizes (*N*), populations with differing *N*_*nb*_/*N*_breeders_ ratios will drift at differing rates and that population ‘type’ captures this ratio.

For mutations that are selectively neutral with regards to the breeding individual’s probability of survival (i.e., those implicated in mutation accumulation), a shift in survivorship from a Type-II to a Type-I, all else being equal, will correspond with an increase in the rate of drift unless this shift also corresponds with an increase in generation time (Fig. 4a). A corresponding increase in generation time reduces the rate of drift for Type-I populations. Going from a Type-II to Type-I population, the ‘pace’ of life decreases as life expectancy increases, but the ‘shape’ steepens indicating an increase in the strength of ageing (Baudisch 2011, see Fig. 1b, c). This shift in population from Type-II to Type-I, therefore, corresponds to an increase in senescence. With Type-I populations, unlike Type-II, the contribution that mutation accumulation makes to senescence does appear to be sensitive to generation time. It follows that Type-I populations with younger generation times will experience drift at a greater rate than those with older generation times and hence a greater contribution of mutation accumulation to senescence is expected. Considering the transition from a pre-industrial/hunter-gatherer human population to a modern-industrialised population, this also results in a shift towards slower pace and steeper shape (Fig. 1). However, the drift simulations suggest that, in terms of the rate of the heterozygosity loss, this shift does not correspond to a marked change in the contribution mutation accumulation may make to senescence. Although the pace/shape metrics used to tease apart how fast and how strongly populations senesce indicate an almost linear change from Type-II to pre-industrial/hunter-gatherer to modern populations (Fig. 1b, c), our results suggest that the contribution of mutation accumulation to senescence is dependent upon population type and, for Type-I populations, this is also dependent upon generation time. Modern human populations may have ‘pace’ and ‘shape’ metrics distinct from pre-industrial/hunter-gatherer populations, but both Type-I populations drift at similar rates and are equally sensitive to generation time (Fig. 4).

The relationship *s* > 1/2*N*_*e*_ describes the conditions required for selection to dominate drift in allele frequency evolution. However, as we have shown, the relative rates of drift between age-structured populations do not always correspond to the relative effective sizes. Figure 5a shows that when negative selection (*s* = 0.01) is experienced, the rate at which the mutation is lost from the populations still bears the hallmarks of the differential rates of drift between population types. For the older breeding populations (T + 20), the Type-II and Hadza populations have noticeably distinct distributions of heterozygosity values at time point *t* = 1000 (Fig. 5b). With strong selection (e.g., *s* = 0.1), as predicted, selection dominates the loss of heterozygosity and all three populations evolve at a similar rate and with similar sensitivity to generation time (result not shown). Hence, unless selection is strong relative to 1/2*N*_*e*_, population type will continue to influence the allele frequency distribution of detrimental alleles.

Our treatment of life-table, actuarial senescence is possibly naive in that it simply relates to the increased mortality with age, but is nevertheless consistent with a large body of literature drawn upon for this study (e.g., Baudisch 2011; Wrycza et al. 2015; Colchero et al. 2016). More sophisticated analyses of senescence, based upon detailed life history data, have arisen that move away from this traditional life-table approach where, for example, generation time was identified as capturing the ‘speed of living’ and to be tightly associated with the onset and rate of senescence (Jones et al. 2008). One explicit metric of this is the ratio of fertility rate to age at first reproduction (F/α). Because α is proportional to generation time (*T*), this ‘pace of life’ metric slows with increasing *T*. This result is consistent with ours, although we consider this in terms of the rate of drift. Metrics such as F/α are valuable in predicting the rate of senescence, but they do not tease apart the genetic underpinnings of senescence. We show that a population’s sensitivity to drift and generation time is a function of its ‘type’, which in turn relates to the relative importance of mutation accumulation as a cause of senescence.

Our focus in this paper has been on the contrast between Type-I and Type-II survivorship curves. It remains to be explored how variation in the reproductive distribution (other than just generation time) influences drift. Although the reproductive distribution employed throughout this manuscript corresponds well with modern and pre-industrialised human populations, the chimpanzee reproductive distribution is quite different as it spans their entire adult life (Bronikowski et al. 2016). Fitting a modern reproductive distribution to a Type-II population is likely to be an unrealistic contrivance and in need of further investigation. Nevertheless, the general principle that manipulating survivorship and generation time can influence the relative contribution of mutation accumulation to senescence opens up the possibility of laboratory investigations that could have some bearing on the evolution of human senescence.

### Data archiving

The R Script and input data are available from https://github.com/AndyOverall/DriftAgeStruct, along with GNU public license details.
