# Correction: Population type influences the rate of ageing

**DOI:** 10.1038/s41437-019-0222-2

**Published:** 2019-05-07

**Authors:** Andrew DJ. Overall, Richard GA. Faragher

**Affiliations:** School of Pharmacy and Biomolecular Sciences, Huxley Building, University of Brighton, Brighton, East Sussex BN2 4GJ UK

**Keywords:** Evolutionary genetics, Population genetics

**Correction to: Heredity;**

10.1038/s41437-019-0187-1; published online 08 February 2019

This article was originally published under standard License to Publish, but has now been made available under a CC BY 4.0 license. The PDF and HTML versions of the paper have been modified accordingly.

